# Do we know enough to recommend corticosteroids in acute respiratory distress syndrome?

**DOI:** 10.1186/s13054-017-1920-x

**Published:** 2017-12-28

**Authors:** Mathieu Blot, Arnaud Salmon-Rousseau, Pascal Chavanet, Lionel Piroth

**Affiliations:** Département d’Infectiologie, Centre Hospitalier Universitaire, 14 rue Paul Gaffarel, Dijon, 21079 France

We read with interest the evidence-based recommendations for the use of prolonged corticosteroids in early moderate to severe acute respiratory distress syndrome (ARDS) published in *Critical Care Medicine* by the Corticosteroid Guideline Task Force of SCCM and ESICM [1]. Whether corticosteroids have to be administered in adult patients with ARDS remains a matter of debate. Based on nine randomized controlled trials (RCTs), including eight RCTs selected in the meta-analysis of Meduri et al. [2] and one additional recent RCT [3], the task force found that the use of prolonged corticosteroids in early ARDS has a beneficial effect on in-hospital mortality (RR 0.64, 95% CI 0.46 to 0.89) [1]. However, several caveats and limitations must be pointed out.

First, two trials from the meta-analysis (Rezk 2013, Sabry 2011), which were not found in the Medline/PubMed database, are at high risk of bias (especially concerning the blinding of participants and staff), and two others (Confalonieri 2005, Liu 2012), which show impressive results, are questionable [2]. Indeed, in the Confalonieri study, delayed septic shock was far more often observed in the placebo group (52%) than in the hydrocortisone group (0%). It is unlikely that such a difference can be attributed to a beneficial effect of hydrocortisone in light of the results of the HYPRESS multicenter trial that showed that hydrocortisone did not prevent progression from severe sepsis to septic shock [1, 4]. In the Liu study, there was a major attrition bias since the placebo group showed markedly significant higher baseline arterial lactate concentrations.

As a result, Ruan et al.’s meta-analysis, which did not include these four small trials, found that corticosteroids in ARDS did not improve longer-term outcomes and even more could be harmful in certain subgroups, such as influenza-related ARDS [5].

In addition, corticosteroids did not seem to have significant side effects other than hyperglycemia. However, it can be speculated that the rate of side effects should be higher in a real-life population of ARDS, as patients with immunodepression, uncontrolled diabetes or at high risk of side effects were not included in these trials.

Considering all these points, we believe that the recommendations of the task force for adjunctive corticosteroids in ARDS are based on insufficient evidence, and at least should be limited to the subset of patients who could have been included in the supportive trials. It thus seems rather prudent to wait for the results of the ongoing multicentric trials evaluating this strategy.

## Abbreviations

ARDS: Acute respiratory distress syndrome
RCT: Randomized controlled trial

## References

[1] Annane D, Pastores SM, Rochwerg B (2017). Guidelines for the diagnosis and management of critical illness-related corticosteroid insufficiency (CIRCI) in critically ill patients (part I): Society of Critical Care Medicine (SCCM) and European Society of Intensive Care Medicine (ESICM) 2017. Crit Care Med.

[2] Meduri GU, Bridges L, Shih M-C (2016). Prolonged glucocorticoid treatment is associated with improved ARDS outcomes: analysis of individual patients’ data from four randomized trials and trial-level meta-analysis of the updated literature. Intensive Care Med.

[3] Tongyoo S, Permpikul C, Mongkolpun W (2016). Hydrocortisone treatment in early sepsis-associated acute respiratory distress syndrome: results of a randomized controlled trial. Crit Care.

[4] Keh D, Trips E, Marx G (2016). Effect of hydrocortisone on development of shock among patients with severe sepsis: the HYPRESS randomized clinical trial. JAMA.

[5] Ruan S-Y, Lin H-H, Huang C-T (2014). Exploring the heterogeneity of effects of corticosteroids on acute respiratory distress syndrome: a systematic review and meta-analysis. Crit Care.

